# Influence of Implant Material and Surface on Differentiation and Proliferation of Human Adipose-Derived Stromal Cells

**DOI:** 10.3390/ijms19124033

**Published:** 2018-12-13

**Authors:** Susanne Jung, Lauren Bohner, Marcel Hanisch, Johannes Kleinheinz, Sonja Sielker

**Affiliations:** Department of Cranio-Maxillofacial Surgery, Research Unit Vascular Biology of Oral Structures (VABOS), University Hospital Muenster, 48149 Muenster, Germany; Susanne.Jung@ukmuenster.de (S.J.); Lauren.Oliveiralimabohner@ukmuenster.de (L.B.); Marcel.Hanisch@ukmuenster.de (M.H.); Johannes.Kleinheinz@ukmuenster.de (J.K.)

**Keywords:** hADSC, tissue regeneration, implants, titanium, zirconia

## Abstract

For the guided regeneration of periimplant hard and soft tissues, human adipose-derived stromal cells (hADSC) seem to be a promising source for mesenchymal stromal cells. For this, the proliferation and differentiation of hADSC were evaluated on titanium and zirconia dental implants with different surface treatments. Results were compared to edaphic cells as human osteoblasts (hOB) and human gingival fibroblasts (HGF). Primary cells were cultured on (1) titanium implants with a polished surface (Ti-PT), (2) sandblasted and acid-etched titanium (Ti-SLA), (3) sandblasted and alkaline etched zirconia (ZrO_2_-ZLA) and (4) machined zirconia (ZrO_2_-M). The cell proliferation and differentiation on osteogenic lineage were assessed after 1, 7 and 14 days. Statistical analysis was performed by one-way ANOVA and a modified Levene test with a statistical significance at *p* = 0.05. PostHoc tests were performed by Bonferroni-Holm. Zirconia dental implants with rough surface (ZrO_2_-ZLA) showed the highest proliferation rates (*p* = 0.048). The osteogenic differentiation occurred early for zirconia and later for titanium implants, and it was enhanced for rough surfaces in comparison to polished/machined surfaces. Zirconia was more effective to promote the proliferation and differentiation of hADSCs in comparison to titanium. Rough surfaces were able to improve the biological response for both zirconia and titanium.

## 1. Introduction

The treatment of periimplant bone defects usually requires a reconstructive surgical procedure, with autogenous bone or biomaterial-based bone grafting to stimulate bone regeneration [1,2,3]. Despite the favorable clinical results showed by these techniques, disadvantages arise, such as high morbidity, limited availability and risk of infection [2,3,4].

Bone regeneration pathway involves the proliferation and differentiation of mesenchymal stem cells into the osteogenic lineage [5]; thus, bone tissue engineering makes it a suitable option to treat periimplant defects. The application of osteogenic cells in combination with physiological components involved on bone remodeling have shown improved outcomes in comparison to biomaterials alone [3].

A promising source for the stem cells is the adipose tissue, since it is easily collected using a low invasive approach. Human adipose-derived cells (hADSCs) differentiate into osteogenic and angiogenic lineage and represent for potential bone regeneration [6]. Additionally, extrinsic and intrinsic osteogenic inducers are recommended to optimize the culture environment and increase the cell regeneration potential [7,8]. Nonetheless, the imperative need of this osteogenic inducer is unclear and the efficacy of osteogenic differentiation without the use of an osteogenic medium has been assessed [9].

The cell biological response in periimplant defects is influenced by the dental implant material and surface [10,11,12,13]. Hempel et al. [10] showed a greater cell proliferation and differentiation on zirconia than titanium. A rough surface improved and accelerated the osteogenic response, by increasing the surface area to mimic the bone morphology [11,12,13,14,15].

Previous studies showed the osteoinduction and osteoconduction capability of hADSCs [16,17], however, the influence of the dental implant material and surface treatment has not yet been well established. The present study evaluated the proliferation and differentiation of hADSCs over titanium and zirconia with various surface treatments. The hADSCs were cultured without inducing factors and their biological response was evaluated using human primary osteoblasts (hOBs) and human gingiva fibroblasts (HGF).

## 2. Results

### 2.1. Cell Viability

**Figure 1:**
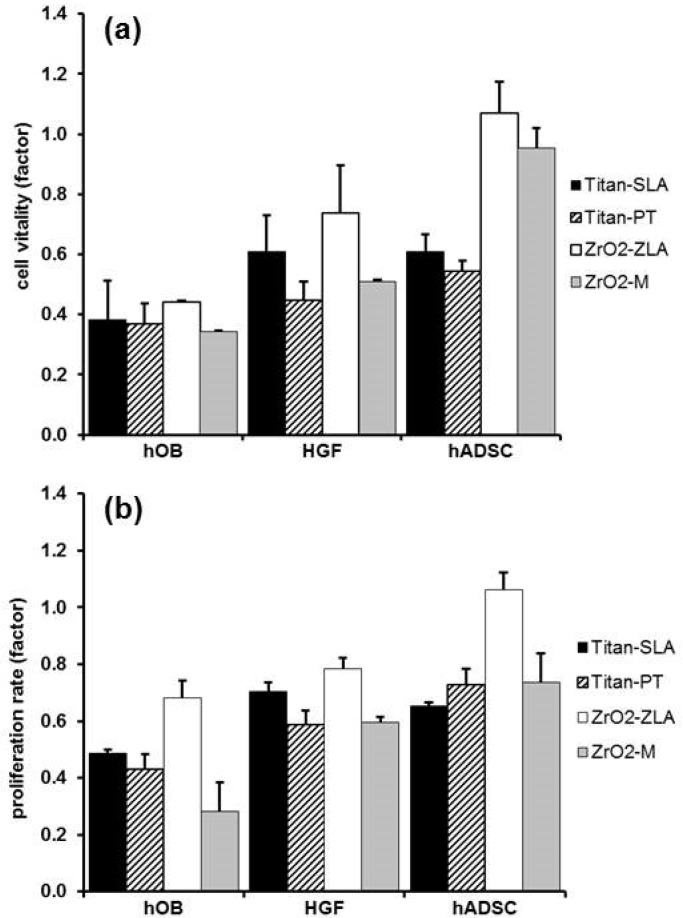

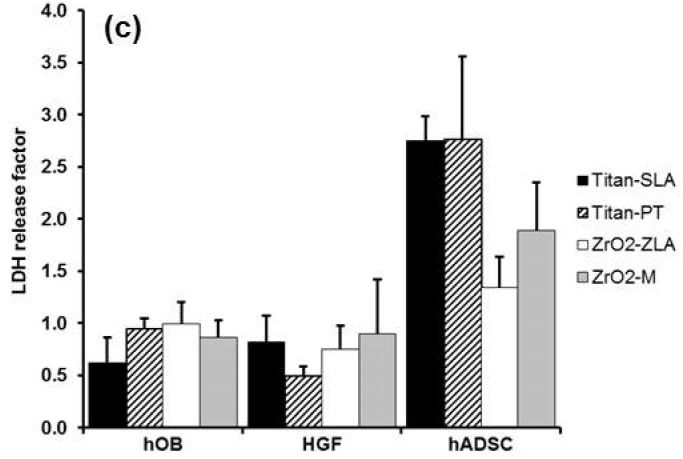
Cell Viability at day 14 correlated to control for human osteoblasts (hOB), human gingival fibroblasts (HGF), and human adipose-derived stromal cells (hADSC) (**a**) cell vitality *p* = 0.021; (**b**) proliferation rate, *p* = 0.041, (**c**) LDH release factor, *p* = 0.0016; standard abbreviation as error marks.

Cell viability among experimental and control groups after 14 days is shown in Figure 1. Zirconia dental implants with a rough surface showed the highest vitality (*p* = 0.022) and proliferation rates (*p* = 0.041). Rough surfaces showed better results compared to polished or machined surfaces. Titanium implants showed higher cytotoxicity than zirconia dental implants (*p* = 0.0016), although minor, and did not affect the cell viability.

### 2.2. Expression of Stem Cell and Osteogenic Marker

**Figure 2:**
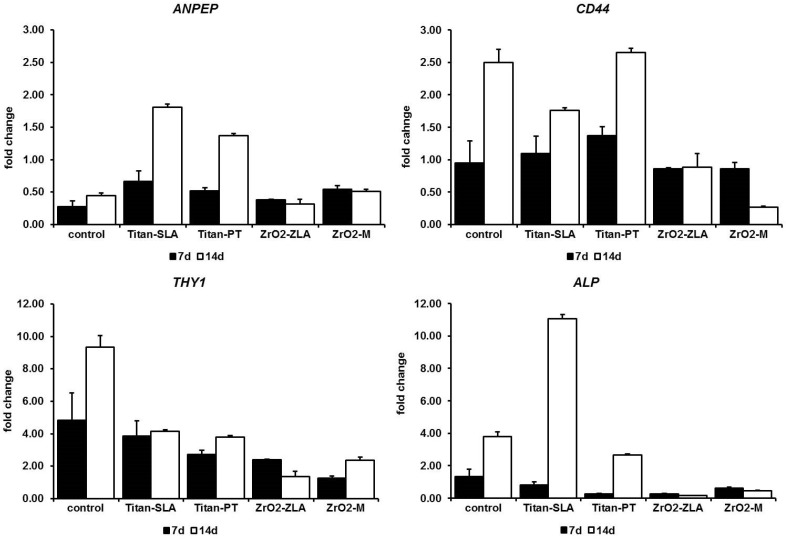
Fold change of stem cell marker compared to day 1 (*ANPEP p* = 0.038, *CD44 p* = 0.18, *THY1 p* = 0.004, *ALP p* = 0.17; standard abbreviation as error marks).

Figure 2 shows the change in stem cells markers compared to day 1 (*p* = 0.04). Gene expression of stem cell markers differed strongly between zirconia and titanium implants (PostHoc analysis *p* = 0.017). For zirconia, gene expression was lower and decreased from day 7 to day 14. In contrast, for titanium and the control group, gene expression was higher and increased from day 7 to day 14 (Figure 2).

**Figure 3:**
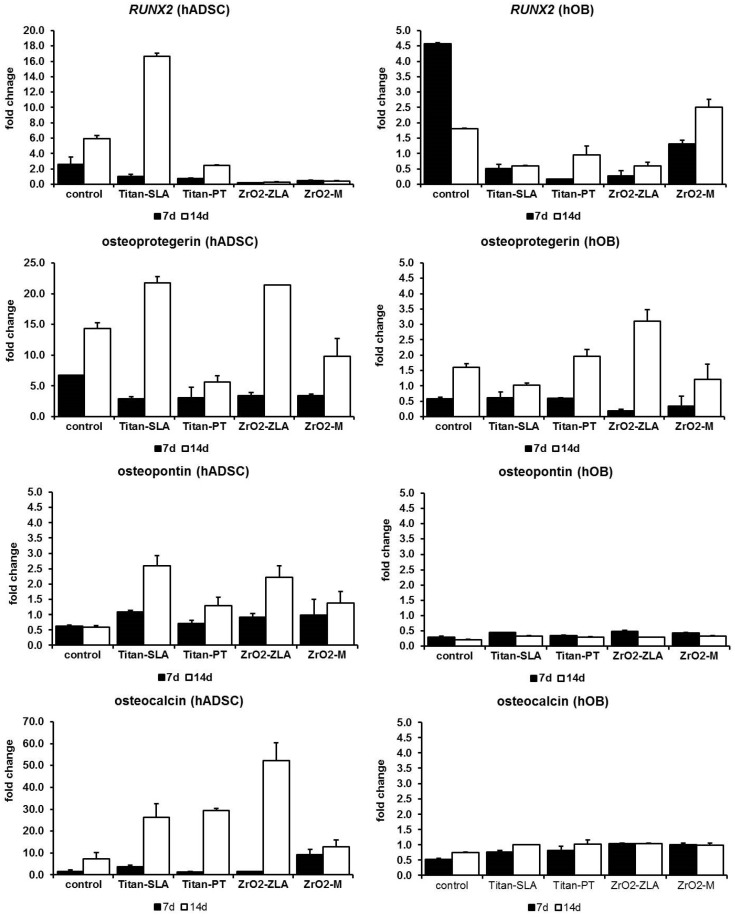
Fold change of osteogenic markers compared to day 1 (*RUNX2 p* = 021 for hADSC, *p* = 0.135 for hOB; osteoprotegerin *p* = 0.011 for hADSC, *p* = 0.008 for hOB; osteopontin *p* = 0.07 for hADSC, *p* = 0.024 for hOB, osteocalcin *p* = 0.024 for hADSC, *p* = 0.25 for hOB; standard abbreviation as error marks).

Figure 3 shows the change of osteogenic marker compared to day 1 for hADSC (*p* = 0.0083) and hOB (*p* = 0.022). Gene expression of *RANKL* was not detectable in any sample. Expression of osteogenic markers did not show a consistent picture, unlike the stem cell marker. In hOB, expression of *RUNX2* and osteoprotegerin increased from day 7 to day 14 with a low fold change, and expression of osteopontin remained constant (Figure 3). In hADSC, expression of osteogenic marker increased strongly and fold change was stronger on rough surfaces except for *RUNX2* on zirconia dental implants. Changes in expression of osteocalcin were remarkable, because no dexamethasone was added to hADSC. Expression of osteocalcin increased strongly, whereas the expression in hOB remained constant (Figure 3).

## 3. Discussion

Zirconia implants showed higher cell viability than titanium implants. From all groups, zirconia dental implants with a machined surface showed the greatest metabolic activity and differentiation. The potential of zirconia to stimulate the osteogenic differentiation was confirmed by the decrease of stem cell markers over time, on rough and machined surfaces, which indicated the trans-differentiation into osteogenic lineage. This effect was not observed for the titanium implants. Similar findings have been reported by Hempel et al. [10], who showed that the metabolic activity was significantly greater for zirconia than for titanium implants 24 and 48 h after plating. Zirconia dental implants seem to be capable of inducing cell proliferation and accelerating the differentiation toward osteogenesis.

The findings of the present study showed that *RUNX2* (the master regulator of osteoblast differentiation) was early expressed in hADSCs over zirconia surfaces. Expression of *RUNX2* takes place at an early stage of osteogenic differentiation, followed by the inhibition of the process at later stages [18]. As the expression of *RUNX2* was not significant for zirconia dental implants after 14 days, it was assumed that the differentiation occurred predominantly during the first 24 h, and remained constant on the following days.

According to Carinci et al. [19], zirconia materials are correlated with an early gene expression, which occurs within the first 24 h of stimulation. Similar findings were shown by Altmann et al. [20], whose osteogenic marker *RUNX2* had higher levels on zirconia after 1 and 7 days. The authors claimed that this response was not only time-dependent, but a function of biomaterial. This hypothesis was confirmed in the present study, since the differentiation of hADSCs was different between titanium and zirconia implants. The titanium implants showed a late response for cell differentiation, resulting in a higher change on the expression of osteogenic markers after 14 days. The late response of titanium to cell viability and differentiation in comparison to zirconia implants had already been shown in previous studies [10,21].

The same hypothesis may be applied to the surface treatment [22]. In this study, rough surfaces showed an early expression of osteogenic markers compared to smooth surfaces. The expression of osteoprotegerin, a receptor related with the osteoclastogenesis [23], was higher on rough surfaces and lower on machined and polished surfaces. Osteogenic markers showed similar results.

Regarding to the use of inducing factors, the expression of osteocalcin did not significantly change over time for osteoblasts with inducing media, whereas for hADSCs, it decreased after 14 days without the use of inducing factors. Thus, albeit the expression of osteogenic markers may be accelerated when an inducing media is used, it seems not to be essential for the osteogenic differentiation.

Previous studies have been controversial regarding the role of inducing factors on osteogenesis. Cecchinato et al. [14] showed the osteogenic differentiation of hADSCs cells on titanium surfaces after 2 weeks; however, Abagnale et al. [21] showed no differentiation of hADSCs without osteogenic medium. For Faia-Torres et al. [9], the osteogenic differentiation only occurred with rough surfaces [9]. Thus, although the findings of this study suggested that material and surface treatment alone may induce the osteogenic differentiation, additional researche is required to confirm this hypothesis.

## 4. Materials and Methods

### 4.1. Study Design and Ethical Approval

The study evaluated the cell viability of hADSCs, primary human osteoblasts (hOB) and human gingiva fibroblasts (HGF) on titanium and zirconia with polished/machined and rough surfaces. The differentiation into osteogenic lineage was assessed for hADSCs and hOBs. The cell viability was assessed based on cell vitality, proliferation and cytotoxicity, whereas the differentiation in osteogenic lineage was measured using on the protein expression analysis. The experiment was designed according to the “Declaration of Helsinki” and approved by the Ethics Committee of the Faculty of Medicine, University of Muenster (#2016-624-f-S, 07 December 2016). Previous to the cell isolation, a written informed consent was obtained from all donors.

### 4.2. Isolation of Primary Human Cell Cultures

All cell colleting procedures were performed anonymously and under sterile conditions. Human fat tissue was collected from leftover tissue of patients who had undergone elective abdominal surgery at the General and Visceral Surgery, University Hospital, Muenster. Oncological surgeries were not included. Human spongiosa and gingiva samples were obtained from the leftover tissue of patients treated at the Department of Craniofacial Surgery, University Hospital Muenster.

Isolation and culture techniques of human fat tissue, primary human osteoblast and human gingiva fibroblasts cells were performed as described previously [6]. Details about the culture medium are described in Table 1. The cells were cultivated in a 5% CO_2_ humidified atmosphere at 37 °C, being fed every 2–3 days and passaged with 10,000 cells/cm^2^ after reaching 90% of confluence.

### 4.3. Main Cell Culture

Cells were cultured on various titanium and zirconia discs (Straumann, Basel, Switzerland) measuring 5 mm diameter, the groups were: (1) titanium implants with polished surface (Ti-PT); (2) sandblasted and acid-etched titanium (Ti-SLA); (3) sandblasted and alkaline etched zirconia (ZrO_2_-ZLA) and (4) machined zirconia (ZrO_2_-M). For hADSCs, no inducing factor was used, whereas the hOB culture was supplemented by an osteogenic medium. The samples were placed in 48-well cell culture plates (Greiner Bio One; Bad Nenndorf, Germany) with a 5% CO_2_ humidified atmosphere at 37 °C. Culturing medium was replaced every 2–3 days during cell culture study. In addition, cells for control group were cultivated as a monolayer in 48-well cell culture plates. Samples were analyzed 1, 7 and 14 days after the beginning of the experiment. Cell culture part was repeated three times.

### 4.4. Cell Viability

The living cell count was performed with the LUNA II system (Logos Biosystems Inc., Villeneuve d’Ascq, France). Proliferation rate was estimated with an in-house MTT assay, which determines the metabolic activity of vital cells. The conversion of the yellow thiazolyl blue tetrazolium bromide (0.5 mg/mL; Sigma-Aldrich, Hamburg, Germany) to the purple formazan was measured at a wavelength of 570 nm. Cytotoxic effects were determined with the Pierce™ LDH Cytotoxicity Assay (ThermoFisher Scientific; Wesel, Germany). All assays were performed according to manufacture protocols and done in triplicates.

### 4.5. Protein Expression Analysis

For protein expression analysis, cells were lysed with the Pierce™ IP Lysis Buffer (ThermoFisher Scientific, Waltham, MA, USA) according to the manufacture’s protocol. The supernatant was frozen at −80 °C for subsequent assays. To determinate secreted proteins, part of the culturing medium was taken before lysis and frozen at −80 °C. Quantification protein determination was performed with the Pierce™ BCA Protein Assay (ThermoFisher Scientific, Wesel, Germany) according to the manufacture’s protocol. The µQuant reader (BioTek, Bad Friedrichshall, Germany) was used for protein determination and ALP assay. Protein expression analysis was evaluated with enzyme-linked immunosorbent assay (ELISA). The ELISAs are listed in Table 2 (abcam, Cambridge, England). ELISAs were performed according to the manufacture’s protocol. Absorbance was measured at a wavelength of 450 nm with the µQuant reader (BioTek, Bad Friedrichshall, Germany). Protein expression was normalized in two steps. The first step was a correlation to whole protein, and the second step was a correlation to day 1. Changes in expression related to day 1 are shown.

### 4.6. RNA Extraction and Real-Time qPCR

For RNA isolation and purification, an RNeasy Micro Kit (Qiagen, Hilden, Germany) was used and performed according to the manufacture’s protocol. Purity and concentration of the isolated RNA was determined by a spectrophotometric reading (NanoDrop™ 2000; ThermoFisher Scientific, Wesel, Germany). For RT-qPCR, a custom-designed RT2 PCR Array (Qiagen, Hilden, Germany) was used (Table 2). For each sample 50 ng RNA was transcripted in complementary DNA (cDNA) with the RT2 First Strand Kit (Qiagen, Hilden, Germany). A DNase treatment to eliminate existent genomic DNA was part of this step. RT-qPCR was performed with the RT2 SYBR Green ROX pPCR Mastermix (Qiagen, Hilden, Germany) according to the manufacture’s protocol in Eppendorf mastercycler ep realplex 4S (Eppendorf, Hamburg, Germany). One µL cDNA was used as template for each reaction. The PCR protocol was: activation of hot start Taq-polymerase for 10 min at 95 °C, followed by 40 cycles with a denaturation step for 15 sec at 95 °C and an annealing/elongation step for 60 s at 60 °C. A melting curve between 65 and 95 °C with a time ramp of 2 min for 1 °C was connected. The ΔΔCT method was used for data analysis with the RT2 Profiler PCR array data analysis web portal (https://www.qiagen.com/de/shop/genes-and-path-ways/data-analysis-center-overview-page/custom-rt2-pcr-arrays-data-analysis-center, accessed on 28 March 2018). Fold changes related to day 1 are shown.

### 4.7. Statistical Analysis

Statistical analysis of expression factors and protein expression factors was carried out by one-way ANOVA and a modified Levene test with a statistical significance at *p* = 0.05. PostHoc tests were performed by Bonferroni-Holm test (Daniel’s XL Toolbox version 6.53; https://www.xltoolbox.net/).

## 5. Conclusions

Zirconia promoted the proliferation and differentiation of hADSCs more than titanium. Rough surfaces improve the biological response for both zirconia and titanium.

## Figures and Tables

**Table 1:** Culture medium.

Cells	Medium	Culture Formula
Human adipose-derived stromal cells (hADSC)	minimum essential medium–Alpha Eagle (α-MEM) (Lonza, Walkersville, MD, USA)	10% fetal bovine serum, 1% amphotericin B (250 µg/mL), 1% glutamine (200 Mm), 1% penicillin (10,000 U/mL)/streptomycin (10,000 µg/mL) (Biochrom Merck, Berlin, Germany)
Primary human osteoblast (hOBs)	Primary human osteoblast (hOBs)	12% fetal bovine serum, 1% amphotericin B (250 µg/mL), 1% glutamine (200 mM), 1% penicillin (10000 U/mL)/streptomycin (10,000 µg/mL) (Biochrom Merck, Germany). For osteogenic differentiation, 16 ng/mL dexamethasone (Merck Pharma, Darmstadt, Germany) was added to the medium.
Primary human gingiva fibroblasts (HGF)	DMEM medium (high glucose and L-glutamine; Gibco, USA) with ¼ Ham′s F12 nutrient mixtures (Sigma, Hamburg, Germany)	10% fetal bovine serum, 1% amphotericin B (250 µg/mL), 1% penicillin (10,000 U/mL)/streptomycin (10,000 µg/mL) (all Biochrom Merck, Berlin, Germany)

**Table 2:** Used and primer (Qiagen, Hilden, Germany) and enzyme-linked immunosorbent assay (ELISA)-Kits (abcam, Cambridge, England).

Gene/Protein	Primer	Protein Assay/ELISA
**Stem Cell Marker**		
*ANPEP*/CD13	PPH05672A	
*CD44*	PPH00114A	
*THY1*/CD90	PPH02406G	
*alkaline phosphatase*	PPH01311F	
**Osteogenic Marker**		
*RUNX2*	PPH01897C	
*TNFSF11/RANKL*	PPH01048F	
osteoprotegerin		ab189580
osteopontin		ab192143
osteocalcin		ab195214
**Housekeeping Genes**		
*RPLP0*	PPH21138F	
*B2M*	PPH01094E	
*GAPDH*	PPH00150F	
*HPRT1*	PPH01018C	
*ACTB*	PPH00073G	

## Abbreviations

hADSC	Human adipose-derived stromal cells
hOB	Human osteoblasts
HGF	Human gingival fibroblasts
